# Contrasting vascular phenotypes of fatal Bothrops envenoming: Hemorrhagic (*Bothrops atrox*) versus thrombotic (*Bothrops lanceolatus*)

**DOI:** 10.1371/journal.pntd.0014873

**Published:** 2026-11-10

**Authors:** Hatem Kallel, Jonathan Florentin, Guy Roger Lontsi Ngoulla, Fatima Radouani, Kinan Drak Alsibai, Loic Savoyen, Stéphanie Houcke, Dabor Resiere

**Affiliations:** 1 Intensive Care Unit, French Guiana University Hospital, Cayenne, French Guiana; 2 INSERM Santé des Populations en Amazonie, UA17, Université de Guyane, Cayenne, French Guiana; 3 Cardiovascular Research Team (UR5_3 PC2E), University of the French West Indies (Université des Antilles), Fort-de-France, France; 4 Department of Pathology, French Guiana University Hospital, Cayenne, French Guiana; 5 Intensive Care Unit, Martinique University Hospital, Fort-de-France, Martinique, France; Universidad Regional Amazónica IKIAM: Universidad Regional Amazonica IKIAM, ECUADOR

## Abstract

**Background:**

Envenomation by Bothrops species produces mainly consumption coagulopathy and hemorrhage, and exceptionally thrombosis which is characteristic of few species. These vascular complications are driven by distinct venom toxin repertoires. In the Neotropics, *Bothrops atrox* typically induces hemorrhagic vasculopathy through SVMP‑mediated endothelial disruption and venom‑induced consumptive coagulopathy (VICC), whereas *Bothrops lanceolatus* triggers a probable thromboinflammatory syndrome characterized by diffuse arterial and venous thromboses. Comparative clinical descriptions of these divergent vascular phenotypes remain scarce.

**Methodology and principal findings:**

We report two fatal events illustrating the contrasting vascular tropisms of these species. A patient bitten by *B. atrox* in French Guiana developed profound VICC, followed by massive intracerebral hemorrhage. Histopathology revealed SVMP-compatible endothelial disruption with perivascular erythrocyte extravasation. In Martinique, a patient bitten by *B. lanceolatus* presented early coagulation disorders, multifocal cerebral infarctions, myocardial ischemia, and multiorgan failure. Thrombus‑formation kinetics (T‑TAS) showed a time-dependent impairment of thrombus formation. These cases highlight the species‑specific vascular signatures of Bothrops envenoming: SVMP‑driven hemorrhagic vasculopathy for *B. atrox* and probable immunothrombotic vasculopathy for *B. lanceolatus*. Delayed presentation contributed to irreversible vascular injury in both patients. Advanced imaging, coagulation phenotyping, and histopathology are essential for delineating the underlying mechanisms involved.

**Conclusions:**

These two fatal cases illustrate a distinct pathophysiological profile of *B. lanceolatus* envenoming, characterized by thrombotic complications, contrasting with the predominantly hemorrhagic phenotype typically observed in Bothrops envenoming. Early antivenom administration is critical to prevent catastrophic complications.

## Author summary

Snakebite envenoming is an important cause of illness and death in tropical regions. Most Bothrops snakebites cause local tissue injury and bleeding related to venom-induced abnormalities of blood clotting. However, some species can cause distinctive and potentially fatal vascular complications. We describe two fatal cases that illustrate markedly different vascular effects of Bothrops venoms. A woman in French Guiana bitten by *Bothrops atrox* developed severe venom-induced coagulopathy followed by a massive intracerebral hemorrhage. Histopathological examination showed disruption of cerebral vascular integrity, supporting a venom-associated hemorrhagic mechanism. In contrast, a man in Martinique bitten by *Bothrops lanceolatus* developed widespread blood clots involving the cerebral and coronary circulation, followed by multiorgan failure. Serial testing of blood-clot formation showed progressive, time-dependent impairment of thrombus formation, suggesting an evolving disturbance of hemostasis. Both patients presented several hours after the bite and received antivenom after a substantial delay. These cases highlight the predominantly hemorrhagic phenotype of typical Bothrops envenoming and the distinct thrombotic phenotype associated with *B. lanceolatus* envenoming. Early recognition, timely antivenom administration, and close monitoring for coagulation disorders and vascular complications are important for the management of severe Bothrops envenoming.

## 1. Introduction

Snakebite envenomation remains a major public health concern in tropical regions, where delays in access to care and species‑specific venom effects contribute substantially to morbidity and mortality. In the Americas, viperid snakes of the genus Bothrops are responsible for the majority of medically significant envenomings [1,2]. Although these species share common clinical features, their venom composition and pathophysiologic effects vary among species, potentially resulting in distinct clinical phenotypes and severe, occasionally life-threatening, complications.

In the Amazon basin, *Bothrops atrox* is the predominant species and a leading cause of severe envenomation [1,3]. Its venom contains metalloproteinases (SVMPs), serine proteases (SVSPs), and phospholipases A_2_ (PLA_2_s) that induce consumption coagulopathy, endothelial disruption, and direct vascular injury [4,5]. These manifestations are further exacerbated by inflammatory reaction and oxidative stress induced by PLA_2_ and L-amino acid oxydase (L-AAO) [6,7]. Hemorrhagic complications, including intracranial bleeding, are well recognized but remain infrequent and poorly characterized [8].

In contrast, *Bothrops lanceolatus*, endemic to Martinique, is associated with a unique thrombotic phenotype not observed with other Bothrops species [2]. Despite limited local tissue involvement, systemic envenomation may trigger diffuse arterial and venous thromboses affecting cerebral, coronary, and peripheral vessels. The mechanisms underlying this prothrombotic syndrome remain incompletely understood but may involve venom‑induced endothelial activation, platelet dysfunction, and complement pathway disturbances [9].

Fatal neurological complications from Bothrops envenomation are rare but represent the most severe end of the clinical spectrum [8,10]. Comparative descriptions of these divergent vascular syndromes are scarce, particularly in settings where access to advanced imaging, histopathology, and intensive care allows detailed characterization.

We report two fatal cases illustrating the contrasting vascular complications of Bothrops envenomation in the French Caribbean and Amazon regions: massive intracerebral hemorrhage following *B. atrox* envenomation in French Guiana and fulminant thrombotic syndrome following *B. lanceolatus* envenomation in Martinique. To our knowledge, this is the first comparative, tissue-supported description of the two opposing vascular tropisms of the genus Bothrops documented within a single healthcare system.

## 2. Methods

### 2.1. Ethics statement

All data presented in this manuscript are fully anonymized. For Patient 1, verbal informed consent for the use of personal clinical information was obtained and documented in the medical record. In addition, the patient and her relatives received an information leaflet at admission explaining that anonymized clinical data may be used for research purposes, with the option to decline participation. For Patient 2, verbal informed consent for publication was obtained from the patient’s family and recorded in the medical file.

### 2.2. Study design and setting

Patient 1 was managed in the intensive care unit (ICU) of the French Guiana University Hospital (Cayenne), the referral center for snakebite envenoming in French Guiana, where *B. atrox* is the predominant medically significant species [1,3]. Patient 2 was managed in the ICU of the Martinique University Hospital (Fort-de-France), where *B. lanceolatus* is the only medically significant viperid [2]. The report was prepared in accordance with the CARE guidelines for case reports.

### 2.3. Data collection

Demographic, clinical, biological, imaging, therapeutic, and outcome data were prospectively recorded. All time points are expressed relative to the time of the bite (H+). Laboratory parameters were measured in the accredited central laboratories of each hospital using routine methods; normal ranges are those of the local laboratories.

### 2.4. Snake identification

In French Guiana, the snake was identified as *B. atrox* based on the patient’s description (size, triangular head, local vernacular name “grage”), its recognition on a photograph, and the clinical and biological features of envenomation. In Martinique, *B. lanceolatus* is the only venomous snake species endemic to the island; identification was therefore based on the geographic origin of the bite and the compatible clinical presentation [2]. Neither snake was brought to the hospital.

### 2.5. Antivenom therapy

Antivenom was administered intravenously according to the local protocols of each center. In French Guiana, the polyvalent F(ab’)_2_ antivenom Antivipmyn Tri (Instituto Bioclon, Mexico) is used for Bothrops envenoming with signs of systemic or extensive local envenoming [1]. Antivipmyn Tri is a polyvalent F(ab’)_2_ antivenom containing antibody fragments directed against the venom of *B. asper*, *Crotalus durissus terrificus,* and *Lachesis muta*. It is manufactured by Instituto Bioclon, Mexico (Registry N° 58583 SSA IV). The product has a nominative temporary authorization for use (ATU) in France granted by the National Agency for the safety of medicines and Health products (ANSM) (Product ID Code: 3400893189627).

In Martinique, the monospecific F(ab’)_2_ antivenom Bothrofav (MicroPharm Ltd, UK) is administered as early as possible to all patients with signs of envenoming, with repeated doses according to clinical progression [2]. Bothrofav is manufactured by MicroPharm Ltd, a UK-based company, and consists of purified F(ab’)2 fragments of immunoglobulins from the plasma of horses immunized with *B. lanceolatus* venom. The product has a nominative ATU in France (Product ID Code: 3400958910388) granted by the ANSM. Both products are used in France under a nominative temporary authorization for use (ATU) granted by the ANSM.

### 2.6. Imaging and functional investigations

Images and functional investigations were interpreted by senior specialists of each center as part of routine care.

## 3. Results

The clinical courses of the two patients are described below

### 3.1. Patient 1 – Fatal hemorrhagic stroke following *Bothrops atrox* envenoming in French Guiana

A 50‑year‑old woman with no known preexisting medical conditions was bitten at 17:00 on the third toe of the right foot by a snake identified as *B. atrox*. The snake was approximately 60 cm long, had a triangular head, was recognized by the patient as a “grage”, and correspond to a photograph of *B. atrox*. Progressive edema extended to the lateral malleolus. She presented 11 hours later to a remote health center, where a mid‑leg tourniquet was found in place. Prehospital treatment consisted of morphine, paracetamol, nefopam, and metoclopramide. The 20‑minute whole‑blood clotting test was uncoagulable, prompting helicopter transfer to the French Guiana University Hospital in Cayenne.

On arrival (17 hours after the bite), the patient was alert (GCS 15), with a blood pressure of 158/88 mmHg, a heart rate of 53 bpm, a temperature of 36.5 °C, and an oxygen saturation of 100% on room air. She reported recurrent vomiting with blood streaks. Examination revealed a small necrotic‑hemorrhagic lesion of the third toe and edema extending above the lateral malleolus (Fig 1). Laboratory tests revealed a hemoglobin level of 12.1 g/dL, a platelet count of 275 G/L, a creatinine level of 105 µmol/L, an alanine aminotransferase (ALAT) level of 10× normal, a CRP level of 3.1 mg/L, a non‑measurable PT/aPTT, and an undetectable fibrinogen.

**Fig 1:**
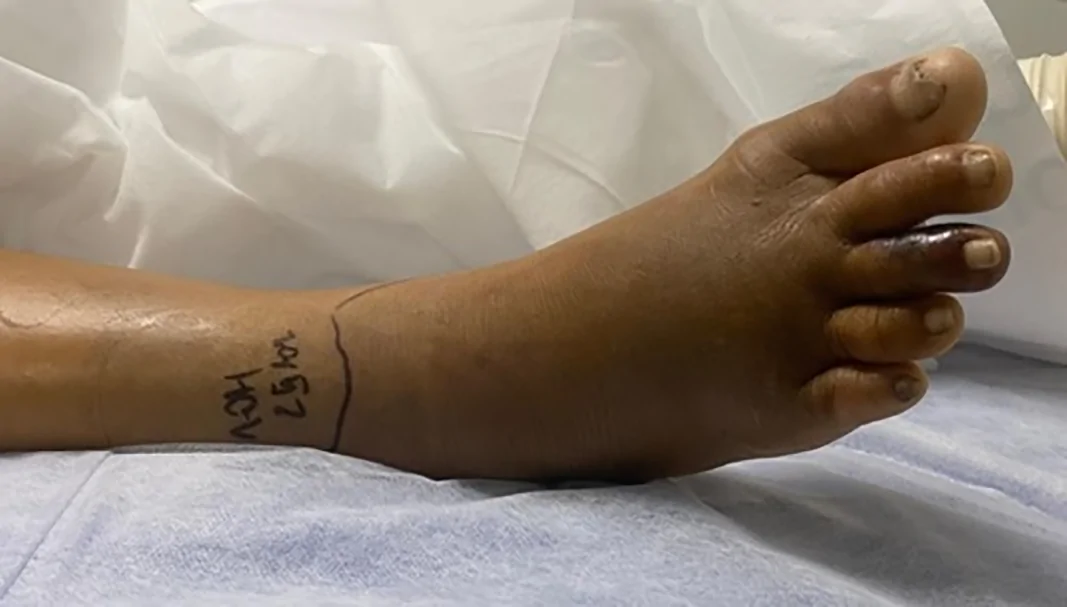
Photograph of the right foot. Necrotic‑hemorrhagic lesion of the third toe and edema above the lateral malleolus.

She received 10 vials of Antivipmyn Tri 18 hours after the bite, corresponding to a stated neutralizing capacity of 7800 LD_50_ of *Bothrops asper* venom in the mouse potency assay. Thirty minutes after the infusion, the patient developed an anaphylactoid reaction characterized by erythematous plaques and oxygen desaturation, which was treated with epinephrine, corticosteroids, and antihistamines.

Shortly thereafter, she developed somnolence, persistent vomiting, left‑sided hemiparesis, facial palsy, and hemispatial neglect. A contrast‑enhanced CT scan performed 24 hours after the bite revealed a massive right frontoparietal intraparenchymal hematoma (82 mm) with active contrast extravasation, associated subarachnoid hemorrhage, mild midline shift, and no arteriovenous malformation. The lesions were more likely attributed to distal frontal artery branch bleeding secondary to venom‑induced coagulopathy rather than aneurysmal rupture (Fig 2). She received two units of fresh frozen plasma and 3 g fibrinogen.

**Fig 2:**
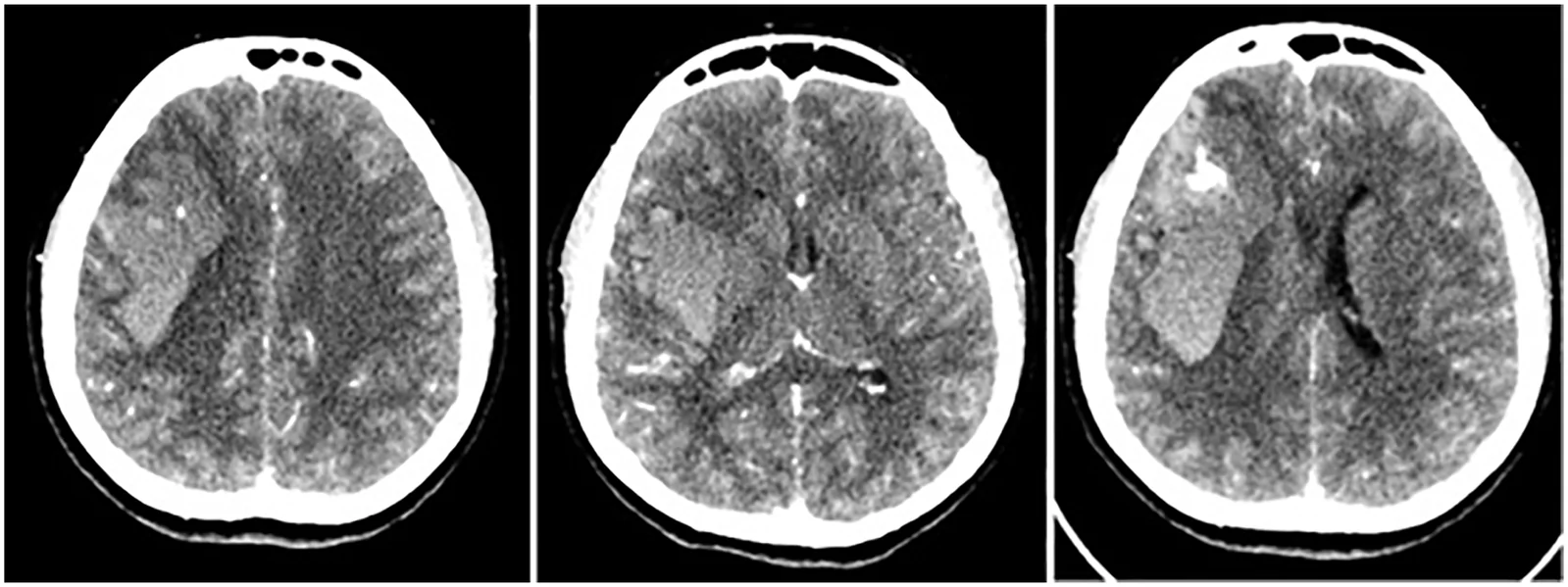
Cerebral contrast‑enhanced CT scan (24 hours after the bite).

During ICU management, blood pressure was controlled with intravenous nicardipine. The coagulation parameters normalized within 35 hours. The creatinine level peaked at 121 µmol/L before improving. The hematocrit decreased to 18.7% with laboratory evidence of hemolysis; the platelet count decreased to 118 G/L.

At 70 hours post-bite, the patient had deteriorated to a GCS score of 4 with anisocoria. CT imaging revealed a stable hematoma volume but a worsening mass effect with a 10‑mm midline shift and temporal transtentorial herniation. Decompressive craniectomy with hematoma evacuation resulted in transient reversal of anisocoria.

Histopathological examination of the brain specimen revealed reactive connective tissue with congested vessels, prominent perivascular extravasation of erythrocytes and neutrophils, and disruption of the CD34-positive endothelial lining, findings consistent with venom-induced vascular injury (Fig 3).

**Fig 3:**
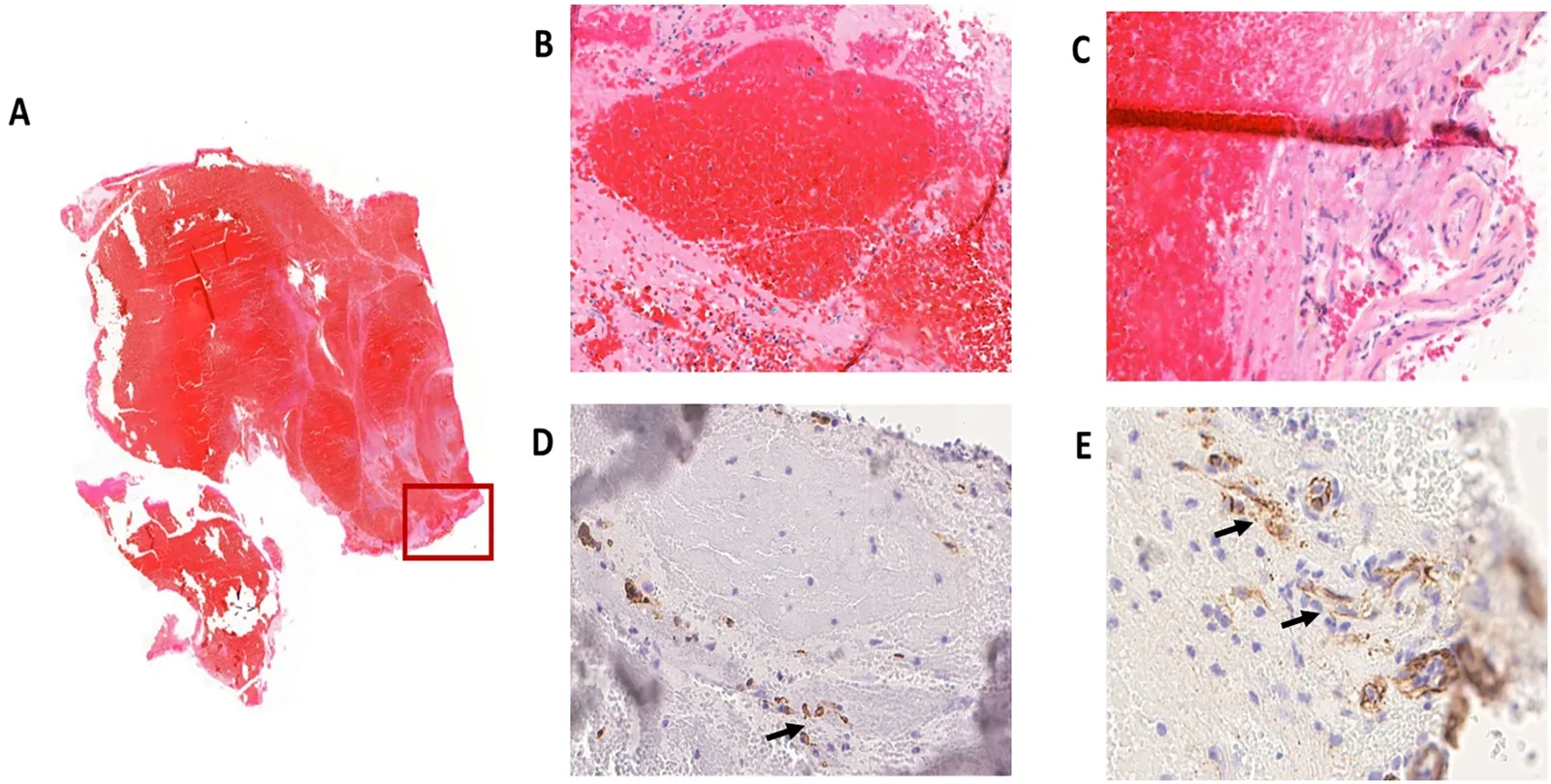
Right frontal intraparenchymal hemorrhage after *B. atrox* envenomation. **Panel A** (hematoxylin and eosin stain) shows a large intraparenchymal hematoma with near-complete replacement of the cerebral parenchyma by extravasated erythrocytes and focal fibrinoleukocytic deposits, which is consistent with acute hemorrhage (×5). **Panels B and C** (hematoxylin and eosin stain) demonstrate, at higher magnification, a thin peripheral rim of reactive connective tissue containing small- and medium-caliber congested vessels, with marked perivascular extravasation of erythrocytes and neutrophils (×200). **Panels D and E** (CD34 immunohistochemical staining) highlight endothelial cells (brown), supporting vascular disruption and loss of vascular integrity (Arrows) (×200). These findings are consistent with an acute hemorrhagic process in the setting of venom-associated vasculopathy and coagulopathy.

Soon after surgery, she developed refractory intracranial hypertension and recurrent anisocoria that progressed to bilateral mydriasis. Repeat CT revealed pneumocephalus, residual hematoma, persistent midline shift, and new intraventricular hemorrhage. Transcranial Doppler revealed reversed flow in the right cerebral circulation, which was consistent with cerebral circulatory arrest. Brain death was confirmed after the withdrawal of sedation.

### 3.2. Patient 2 – Fatal diffuse thrombosis following *Bothrops lanceolatus* envenomation in Martinique

A 45-year-old man with no known preexisting medical conditions was bitten on the right hand at 09:30 while doing landscaping work. The offending snake was *B. lanceolatus*, the only medically significant viperid endemic to Martinique. He presented to a private clinic at 14:00 (04:30 after the bite) with severe pain, edema, and erythema of the right index finger, associated with paresthesia and hypoesthesia of the right upper limb (Fig 4). He was transferred to the University Hospital of Martinique.

**Fig 4:**
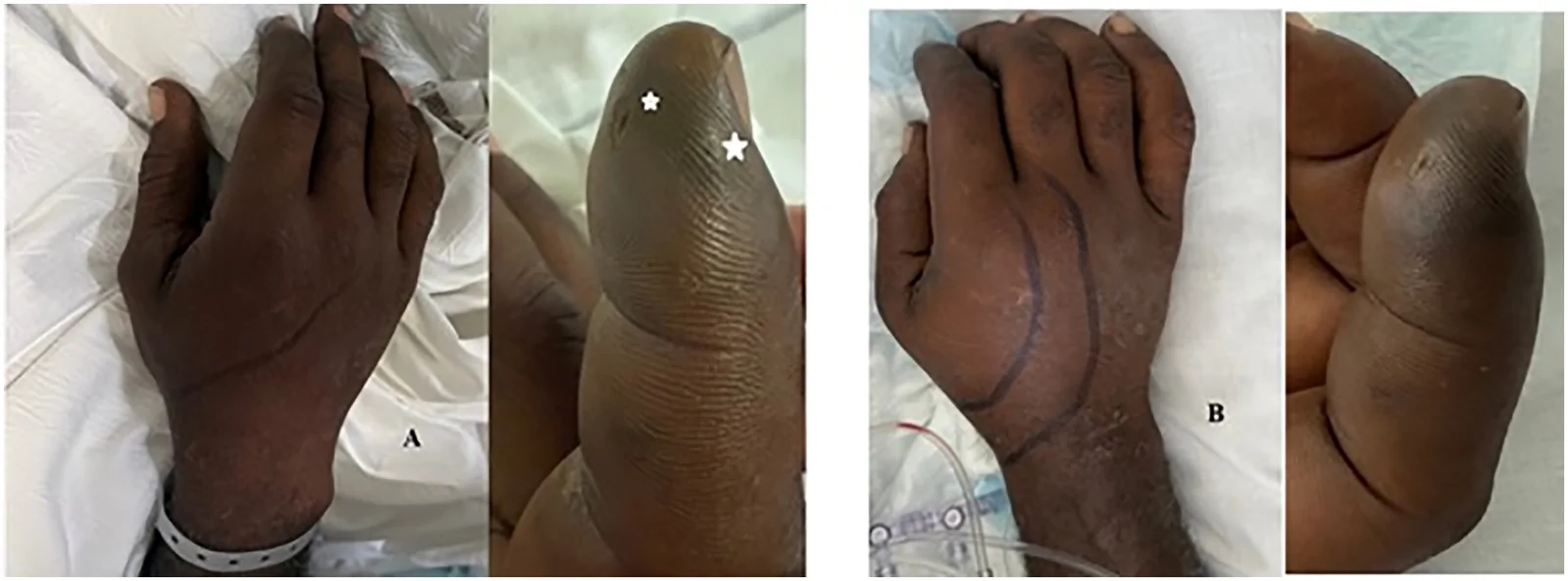
Local signs of envenomation. Panel A shows the clinical appearance at H + 7 after envenomation. The stars indicate the fang marks. Panel B shows the local appearance 48 hours after envenomation, with localized edema of the index finger and thumb of the right hand, without extension to the wrist or forearm.

On arrival at 15:40 (06:10 after the bite), vital signs included a blood pressure of 176/117 mmHg, a heart rate of 95 bpm, a respiratory rate of 16/min, an oxygen saturation of 100%, a temperature of 36 °C, and a GCS score of 13/15. Local findings were limited to edema of the index finger and thenar region. Neurological examination revealed confusion, right‑sided hemiplegia, and right‑sided ptosis. The initial ECG revealed sinus tachycardia, right bundle branch block, and concave ST‑segment elevation in leads II, III, aVF, V5, and V6, associated with tall, peaked T waves (Fig 5). A 30-mL dose of Bothrofav was administered at 16:00 (06:30 after the bite).

**Fig 5:**
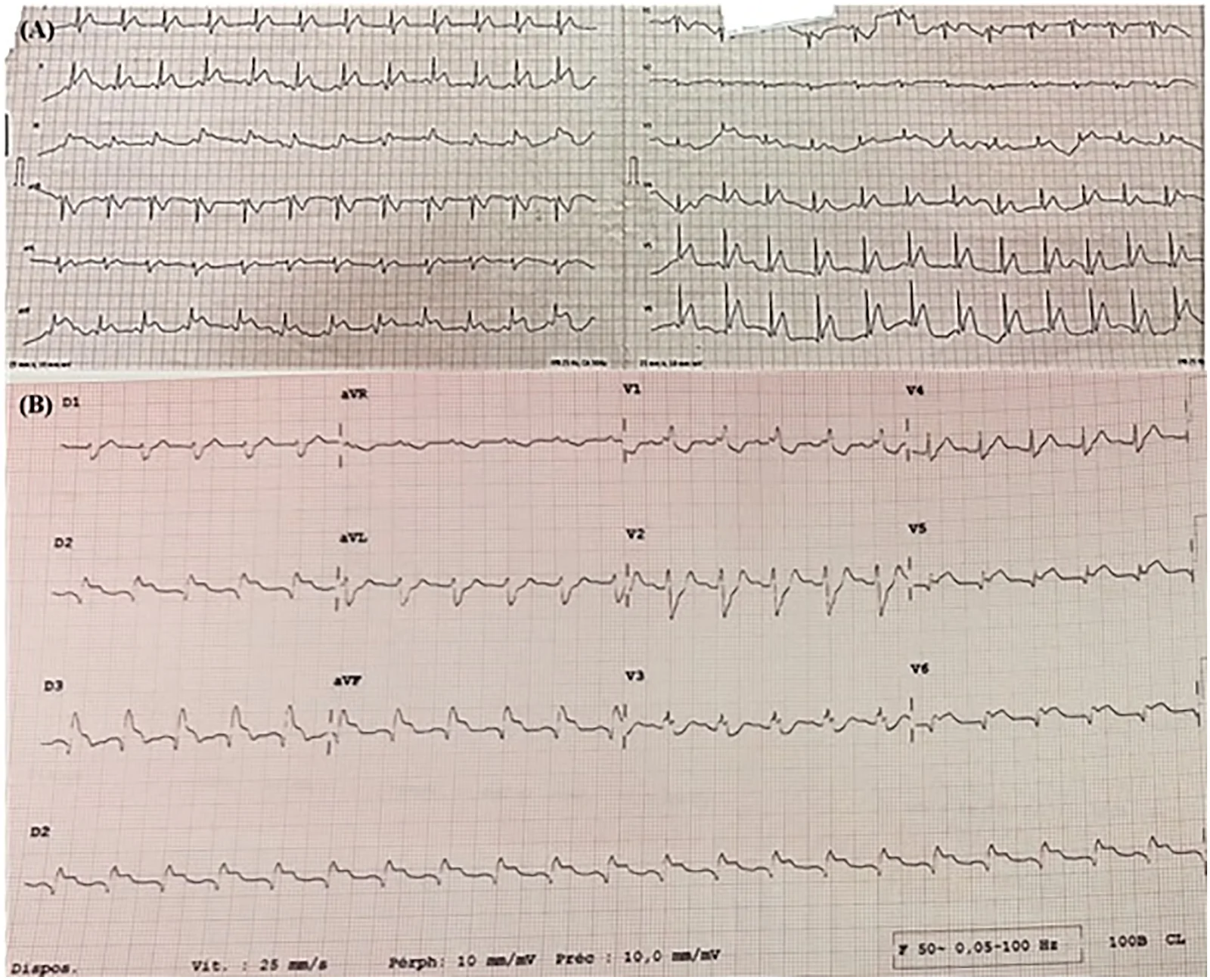
Evolution of Electrocardiographic Findings from Early Repolarization Abnormalities to Acute Inferolateral Myocardial Infarction. Panel A: Electrocardiogram performed at H + 7 showing sinus tachycardia, right bundle branch block, and concave ST-segment elevation in leads II, III, aVF, V5, and V6, associated with tall, peaked T waves. Panel B: Electrocardiogram performed at H + 36 revealing inferolateral ST-segment elevation (pardee waves) involving inferior leads II, III, and AVF, as well as lateral leads V5 and V6, which is consistent with acute inferolateral myocardial infarction.

Initial laboratory tests revealed coagulation abnormalities including thrombocytopenia, elevated troponin I, and increased CK (Table 1). Thrombus‑formation kinetics (T‑TAS) showed rapid microchannel occlusion in control samples, whereas blood obtained from the patient 6 hours after envenomation showed a marked rightward shift, with the thrombus-induced increase in pressure delayed to approximately 20 minutes. At 24 hours, occlusion was further delayed, consistent with sustained, time-dependent impairment of platelet-mediated thrombus formation under arterial shear (Fig 6). Cerebral MRI revealed multiple distal ischemic strokes and probable thrombosis of the left middle cerebral artery (M2), without hemorrhagic transformation (Fig 7).

**Table 1:** Biological parameters recorded during hospitalization.

	Normal range	H7	H13	H18	H24	H36	H42
Platelets count (G/L)	150-400	129	85	40	19	10	73 (transfusion)
PT (%)	>70	67	80	77	78	58	52
Fibrinogen (g/L)	2-4	1.1	–	1.6	1.9	3.5	4.2
FDP (µg/ml)	<5	>120		>120		73	–
D-dimers (µg/ml)	<0.5	40.4	47.7	47.2	6.98		–
Troponin I (pg/ml)	<33	131	10052	23693	48839	497349	550759
NT-Pro-BNP (pg/ml)	n < 300	–	–	1972	–	–	–
CPK (UI/L)	30-200	332	1912	2991	3147	6819	21357
Creatinine (µmol/L)	55-96	70	102	110	162	–	–
LDH (UI/L)	125-220	234		874	930	–	–
Leukocytes count (G/L)	4-10	9.5	16	21.1	17	12.7	16.8
CRP (mg/L)	<5	0.5	7.5	23	50	129	157
Haptoglobin (g/L)	0.75-2	0.72	–	0.59	0.48	–	0.22
Complement C3 (g/L)	0.82-1.85	–	1.30	–	0.88	0.85	0.52
Complement C4 (g/L)	0.15-0.53	–	1.31	–	0.22	0.24	0.15

CK: Creatine kinase, LDH: Lactic dehydrogenase, FDP: Fibrin degradation products

**Fig 6:**
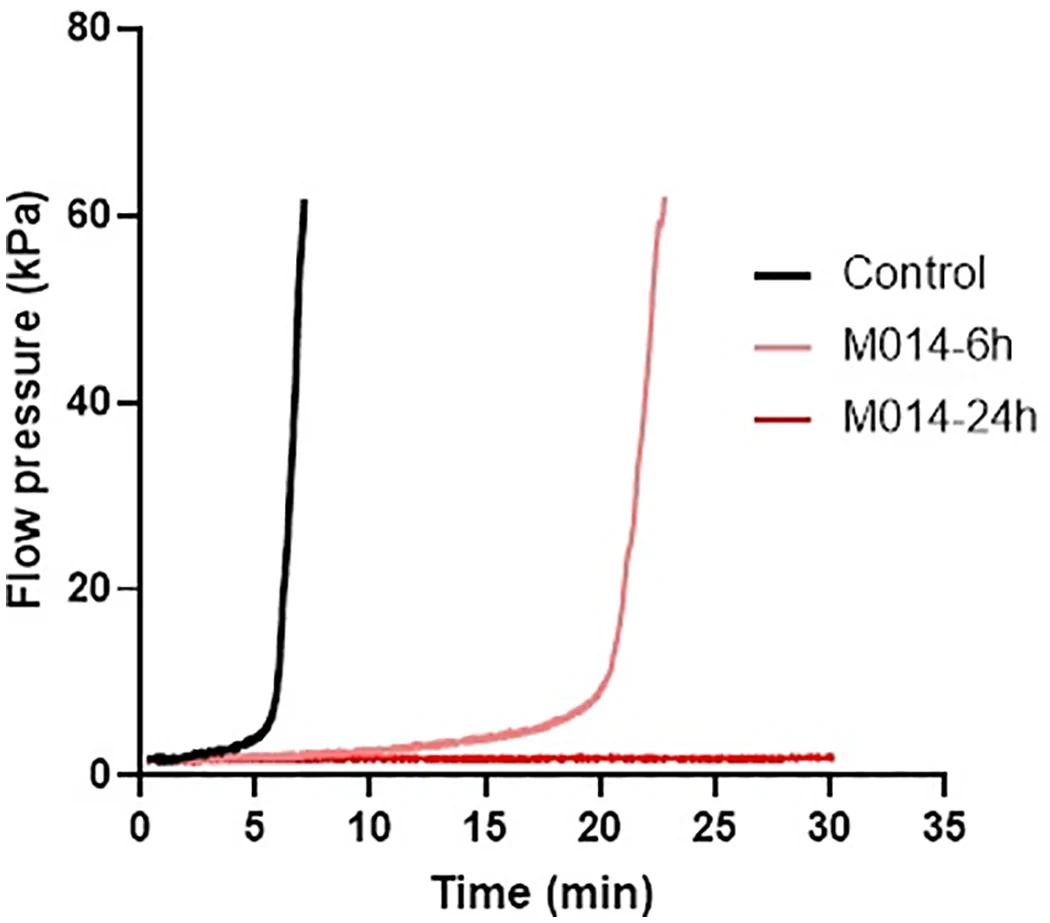
Thrombus formation kinetics assessed via T-TAS after 6 hours (pink curve) and 24 hours (red curve) of bite by *B. lanceolatus* (red curves) vs. a control (black curve).

**Fig 7:**
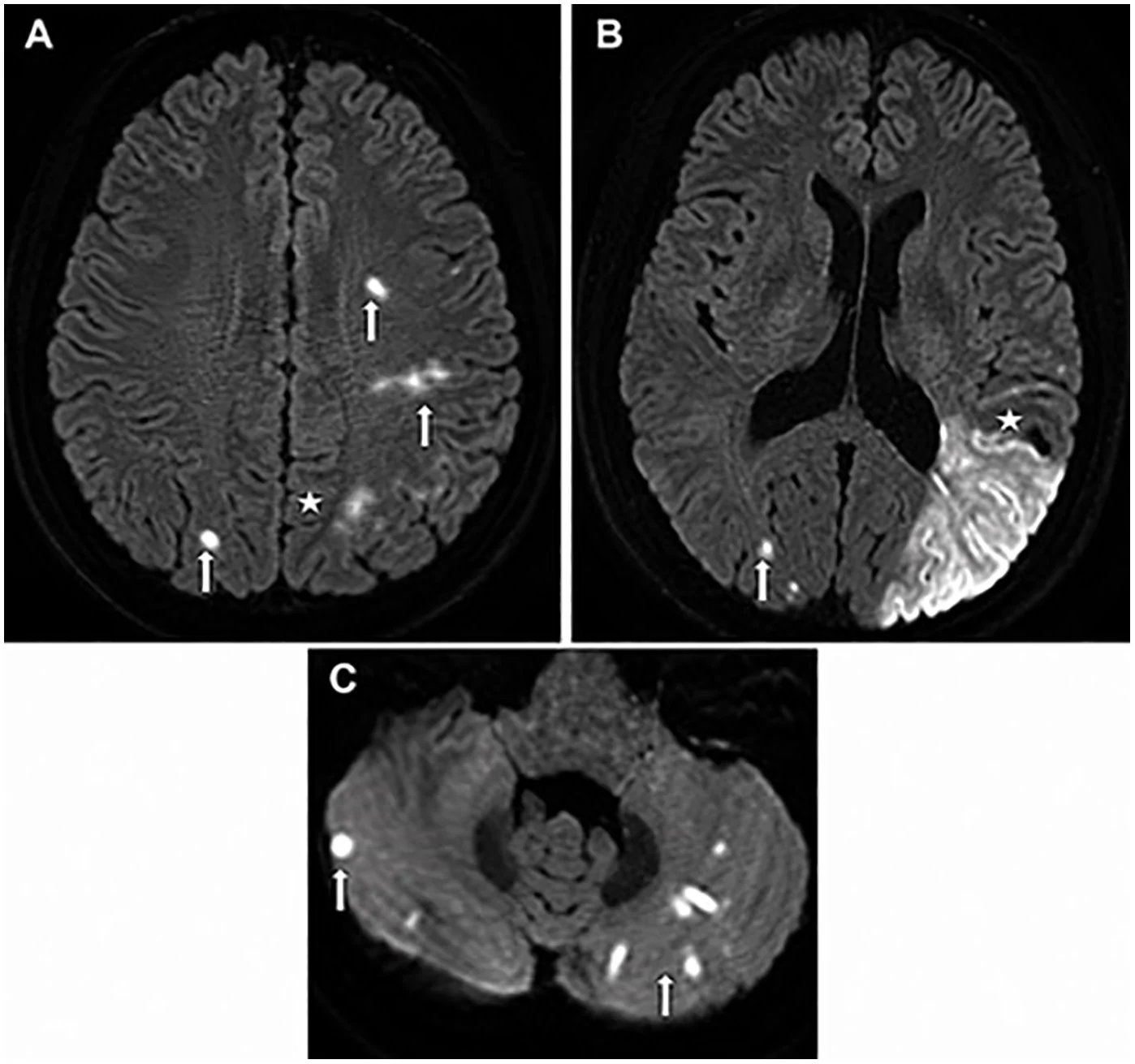
Cerebral MRI diffusion-weighted imaging showing multiple small ischemic strokes in the process of development, with a distal distribution and involving multiple vascular territories, without hemorrhagic transformation, as indicated by white arrows. An evolving ischemic area in the left parieto-occipital and insular regions was also observed, with probable thrombosis of the left middle cerebral artery (M2 segment), indicated by a white star.

In the intensive care unit (ICU), the patient developed acute respiratory failure requiring intubation. At 24 hours, local findings remained limited, and a second 30-mL dose of Bothrofav was administered, corresponding to the dose of antivenom able to neutralize 1800 LD_50_ of *B. lanceolatus* venom in the mouse potency assay. He rapidly developed multiorgan failure, including severe ARDS, hemodynamic instability requiring norepinephrine, anuric acute kidney injury, metabolic acidosis, hyperkalemia, and rhabdomyolysis.

A second ECG at H + 36 revealed inferolateral ST‑segment elevation. Echocardiography revealed regional wall motion abnormalities, biventricular dysfunction, and new severe mitral regurgitation. Laboratory parameters revealed worsening renal failure, escalating rhabdomyolysis, persistent disseminated intravascular coagulation (DIC), and severe thrombocytopenia. Platelet transfusion was administered at H + 42.

On day 2, the patient developed acute hepatocellular failure followed by bilateral fixed mydriasis. Brain death was confirmed after the withdrawal of sedation. He died 48 hours after the bite.

The comparative clinical and biological features of the two fatal cases, illustrating the distinct hemorrhagic and thrombotic vascular syndromes associated with Bothrops envenomation, are summarized in Table 2.

**Table 2:** Comparative characteristics of the two fatal cases of Bothrops envenomation with hemorrhagic and thrombotic vascular complications.

Feature	Case 1 (*B. atrox*)	Case 2 (*B. lanceolatus*)
Delay to hospital	17 h	6 h 10 min
Time from SB to AV	18 h	06 h 30 min
Antivenom	Antivipmyn Tri	Bothrofav
Local severity	Moderate	Mild
Coagulopathy	VICC	DIC with thromboinflammatory phenotype
Main vascular event	Massive intracerebral hemorrhage	Multifocal ischemic stroke + myocardial infarction
Imaging	CT hemorrhage	MRI ischemia
Histology	Endothelial disruption	Not available
Mechanism	Hemorrhagic vasculopathy	Possibly immunothrombosis
Cause of death	Brain herniation	Multiorgan failure + cerebral ischemia

SB: snakebite, AV: antivenom, DIC: disseminated intravascular coagulation, VICC: venom‑induced consumptive coagulopathy.

## Discussion

These two fatal cases illustrate the divergent vascular complications associated with Bothrops envenomation in the Neotropical region and underscore the species‑specific pathophysiological signatures of *B. atrox* and *B. lanceolatus*. Both patients presented to the hospital late and received delayed antivenom. Although local severity was discordant with systemic outcome in both, each patient developed fulminant systemic complications driven by distinct venom-mediated mechanisms: hemorrhagic vasculopathy in French Guiana and diffuse thrombosis in Martinique.

The first patient was bitten by a *B. atrox*, presumably a juvenile, given the description of the snake and the contrast between the mild local tissue effects and the severe systemic hemorrhagic manifestations [11]. She received delayed antivenom and developed severe intracerebral hemorrhage. Hemorrhagic stroke is a severe complication of Bothrops envenomation [8,12], and its pathophysiology is multifactorial. After a bite by a Bothrops snake, Silva De Oliveira et al. documented the presence of venom in the patient’s brain tissue after death [13]. The venom of *B. atrox* contains 101 distinct proteins, comprising predominantly SVMPs (40.2%), PLA_2_s (19.5%), SVSPs (11%), L-AAO (7.3%), C-type lectins (7.3%), and disintegrins (2.4%) [5]. These toxins may exert direct effects, either independently or synergistically, that may contribute to a time- and dose-dependent vascular toxicity [4]. SVMPs, mainly those of the PIII class, can target the cerebral microvasculature, degrading basement membrane components and disrupting endothelial cell–cell adhesion, thereby compromising vascular integrity and rendering microvessels fragile and prone to rupture [4,14–16]. Additional contributions from PLA_2_‑mediated inflammation, oxidative stress, and direct hemorrhagin activity may exacerbate vascular injury [4]. Concurrent VICC, characterized by rapid depletion of fibrinogen and clotting factors, further increases the risk of bleeding. In this case, the presence of active contrast extravasation on CT, in the absence of a vascular malformation or arterial aneurysm, strongly suggested ongoing microvascular hemorrhage consistent with venom‑mediated vascular toxicity, which was likely exacerbated by delayed antivenom administration. Although the available data indicate no significant association between delayed antivenom administration and stroke [8], early antivenom therapy and timely neurocritical care may reduce the risk of neurologic complications after snakebite envenomation [12].

The second case also illustrates the consequences of the delayed management of *B. lanceolatus* envenomation. Despite the availability of an effective antivenom and a universal healthcare system that has, for nearly two decades, successfully reduced mortality and major disability associated with this envenomation, this case underscore the continuing need for prevention strategies and public awareness regarding snakebites [2,17]. Initial management consists of rapid administration of the specific antivenom, Bothrofav, in combination with nonspecific supportive measures. This patient arrived at the University Hospital and received antivenom approximately 7 hours after the bite. Diffuse thromboses and severe coagulopathy were already present upon hospital admission. Neither antivenom administration nor advanced intensive care was sufficient to prevent a fatal outcome. This observation suggests that thromboinflammation may play a role in the pathophysiology of thrombotic complications following *B. lanceolatus* envenomation [9]. *B. lanceolatus* venom contains several protein families, comprising predominantly SVMPs mainly of the PI and PIII classes (41.1%), PLA_2_s (15.7%), SVSPs (11.4%), L-AAO (5.7%), C-type lectins (11.4%), and disintegrins (1.4%) [5]. In our case, serial T-TAS monitoring showed progressive, time-dependent impairment of thrombus formation, with further prolongation of occlusion time at 24 hours compared with 6 hours after envenomation. These findings were consistent with a dynamic, evolving disturbance of hemostasis following *B. lanceolatus* envenomation. Experimental and clinical data suggest that *B. lanceolatus* venom can induce the release of the venom-associated molecular patterns “VAMPs” [9], which may trigger a complex interplay between inflammatory and thrombotic pathways, involving endothelial activation, platelet-leukocyte interactions, complement and inflammatory pathway activation, reduced ADAMTS13 activity, and intravascular fibrin deposition [9,18,19]. In particular, neutrophil activation and interactions with platelets may promote the release of neutrophil extracellular traps (NETs), which have been implicated in immunothrombosis by providing a scaffold for platelet adhesion, fibrin deposition, and microvascular occlusion [20]. These mechanisms may result in tissue edema, microvascular obstruction, and thrombotic microangiopathy [21]. This case suggests that once lesions secondary to this cascade are established, irreversible organ damage may occur, thereby explaining the failure of antivenom therapy and organ support measures.

Together, these cases emphasize that Bothrops envenomation cannot be considered a uniform clinical entity. *B. atrox* and *B. lanceolatus* exhibit fundamentally different vascular tropisms, hemorrhagic versus thrombotic, requiring distinct diagnostic vigilance and therapeutic priorities. Delayed presentation and late antivenom administration are major determinants of poor outcomes.

## Conclusion

These two fatal cases illustrate a distinct pathophysiological profile of *Bothrops lanceolatus* envenoming, characterized by prominent vascular toxicity and thrombotic complications, in contrast to the predominantly hemorrhagic phenotype typically observed in Bothrops envenoming. Despite early intensive care and antivenom administration, both patients experienced irreversible neurological injury and multiorgan failure. Clinicians must recognize these species‑specific vascular profiles and maintain high suspicion for neurological and cardiovascular complications even when local signs are limited. Early antivenom administration remains critical for preventing irreversible vascular injury.
